# Sexually dimorphic genetic architecture of complex traits in a large-scale F_2_ cross in pigs

**DOI:** 10.1186/s12711-014-0076-2

**Published:** 2014-11-06

**Authors:** Leilei Cui, Junjie Zhang, Junwu Ma, Yuanmei Guo, Lin Li, Shijun Xiao, Jun Ren, Bin Yang, Lusheng Huang

**Affiliations:** Key Laboratory for Animal Biotechnology of Jiangxi Province and the Ministry of Agriculture of China, Jiangxi Agricultural University, 330045 Nanchang, China

## Abstract

**Background:**

It is common for humans and model organisms to exhibit sexual dimorphism in a variety of complex traits. However, this phenomenon has rarely been explored in pigs.

**Results:**

To investigate the genetic contribution to sexual dimorphism in complex traits in pigs, we conducted a sex-stratified analysis on 213 traits measured in 921 individuals produced by a White Duroc × Erhualian F_2_ cross. Of the 213 traits examined, 102 differed significantly between the two sexes (q value <0.05), which indicates that sex is an important factor that influences a broad range of traits in pigs. We compared the estimated heritability of these 213 traits between males and females. In particular, we found that traits related to meat quality and fatty acid composition were significantly different between the two sexes, which shows that genetic factors contribute to variation in sexual dimorphic traits. Next, we performed a genome-wide association study (GWAS) in males and females separately; this approach allowed us to identify 13.6% more significant trait-SNP (single nucleotide polymorphism) associations compared to the number of associations identified in a GWAS that included both males and females. By comparing the allelic effects of SNPs in the two sexes, we identified 43 significant sexually dimorphic SNPs that were associated with 22 traits; 41 of these 43 loci were autosomal. The most significant sexually dimorphic loci were found to be associated with muscle hue angle and Minolta a* values (which are parameters that reflect the redness of meat) and were located between 9.3 and 10.7 Mb on chromosome 6. A nearby gene i.e. *NUDT7* that plays an important role in heme synthesis is a strong candidate gene.

**Conclusions:**

This study illustrates that sex is an important factor that influences phenotypic values and modifies the effects of the genetic variants that underlie complex traits in pigs; it also emphasizes the importance of stratifying by sex when performing GWAS.

**Electronic supplementary material:**

The online version of this article (doi:10.1186/s12711-014-0076-2) contains supplementary material, which is available to authorized users.

## Background

Males and females differ in many aspects, from their chromosomes (X vs. Y) to their body morphologies to their reproductive behaviors. In model organisms such as mice and *Drosophila*, many traits ranging from gene expression levels [1] to embryonic development [2] have been reported to show sexual differences. Furthermore, the prevalence and severity of many common diseases in humans, such as type 2 diabetes [3] and asthma [4], also vary between men and women.

The fact that a broad range of complex traits are sexually dimorphic indicates that males and females are characterized by important biological differences, which can modify the effects of genes on complex traits. Understanding sex-dependent differences in the genetic architecture of complex traits can help to generate insights into the evolution of males and females; it can also help in the development of sex-specific medical treatments in humans and breeding objectives in farm animals. Many traits in humans, such as gene expression levels [5], blood metabolite levels [6], and the prevalence of common diseases [7], have been reported to have sexually dimorphic genetic architectures. Similar situations have also been documented in model organisms such as mice (e.g., fat mass [8]) and *Drosophila* (e.g., gene expression [9]), which indicates that sexually dimorphic genetic control of complex traits is common across species. In contrast, the molecular basis of sex-specific genetic regulation of complex traits in pigs remains less explored.

Genome-wide association studies (GWAS) have been used to identify genetic loci that affect complex traits in pigs, such as traits related to body composition [10], hematology [11], and disease susceptibility [12]. In a previous study, we conducted a large-scale White Duroc × Chinese Erhualian F_2_ cross, measured a variety of traits and obtained 60 K genotypes from more than 1000 F_2_ individuals. Using these data, we performed GWAS to identify the loci associated with various traits [13,14]. However, our previous study and most other GWAS in pigs did not explicitly consider the possibility of sex-specific differences in the genetic architecture underlying the traits examined. The associations between single nucleotide polymorphisms (SNPs) and traits were simply assessed using linear models that treated sex as a fixed effect, which could have reduced the power to detect loci that demonstrated sex-specific differences.

In this study, we used a sex-stratified GWAS to examine sex-specific differences in gene regulation using 213 traits in individuals from our large F_2_ pig experimental population. Data were obtained from a total of 921 F_2_ individuals of both sexes. All the male pigs were castrated when they were 90 days old; therefore, the influence of sex hormones was limited in this study. First, we evaluated sex-specific differences in the 213 traits using a linear regression model. Next, by comparing the genome-wide SNP-trait association signatures found in males versus females, we identified loci that displayed sexually dimorphic expression; these loci were associated with a variety of traits related to meat quality, fatty acid composition, food intake, and hematological parameters. This study emphasizes that sex is an important factor that can modify the effects of the genetic variants underlying complex traits in pigs.

## Methods

### Animals

All the pigs used in this study were the product of a large-scale White Duroc × Erhualian F_2_ cross; the design and management of this experimental population have been described elsewhere [15]. Briefly, two White Duroc boars were mated to 17 Erhualian sows to generate the F_1_ pigs. Then, nine F_1_ boars were crossed with 59 F_1_ sows to produce 1912 F_2_ pigs (in six batches). All the F_2_ animals were raised at the Jiangxi Agricultural University experimental farm (Nanchang, China); they were fed *ad libitum* and weaned at the age of 46 days. The male pigs were castrated at the age of 90 days. The castrated males and the females lived in mixed pens and were treated equally throughout the experiment. All the F_2_ pigs were slaughtered at the age of 240 ± 3 days; overall, we measured more than 400 traits in the F_2_ population. We obtained 60 K SNP genotypes from 1000 F_2_ pigs using porcine SNP60 Beadchips (Illumina). The procedures used to phenotype the pigs and obtain the 60 K genotypes have been described in previous publications [13-17]. All animal work was conducted according to the guidelines for the care and use of experimental animals established by the Ministry of Agriculture of China.

### Data used

We investigated 213 traits in the F_2_ pigs produced by a large-scale White Duroc × Chinese Erhualian F_2_ cross. These traits are related to a broad range of characteristics, including growth, fatness, meat quality, hematological parameters, and serum glucose and lipid levels (See Additional file 1: Table S1). For the 213 traits examined, we obtained both phenotypic values and 60 K SNP genotypes for between 217 and 919 pigs; we had both sets of data for at least 100 individuals for both sexes.

### Quality control of 60 K SNP genotype data

The quality control procedure used for the 60 K SNP genotype data and the details of the GWAS are the same as previously described [14]. Briefly, we retained samples with SNP call rates greater than 10% and Mendelian inconsistence rates smaller than 0.05; we kept SNPs with call rates higher than 0.9, minor allele frequencies higher than 0.05, *P* values greater than 10^−6^ for the Hardy-Weinberg equilibrium test and Mendelian error rates smaller than 0.1. These procedures were implemented in Plink v1.07 [18]. In total, 39 454 SNPs and 921 individuals were used in the subsequent analyses.

### Statistical analyses

We log-transformed (log_2_) the phenotypic values of any traits that strongly deviated from normality (Shapiro test *P* value <10^−8^). We assessed the significance of the effects of sex on the 213 traits examined using the *lm* function in R [19]; batch was included as a fixed effect. To evaluate sex-specific differences in the genetic architecture of the 213 traits, we conducted a sex-stratified analysis in which we estimated trait heritability and performed separate GWAS for males versus females. We used the R package GenABEL [20]; batch was included as a fixed effect. Heritability was estimated based on a kinship matrix calculated from the 60 K SNP genotype data and using the *polygenic* function. The associations between the SNP genotypes and the traits were evaluated using mixed linear models (*mmscore* function). As in the previously described GWAS, sex and batch were fitted as fixed effects. We assessed the empirical significance of the difference in heritability between the two sexes using a permutation test [21]: for each trait, we shuffled the gender labels of individuals 1000 times to generate a null distribution of absolute differences in heritability between the two sexes. We evaluated the *P* values of the associations yielded by the GWAS after applying a Bonferroni correction. The suggestive significance level was set to 1/N_snp,_ where N_snp_ was the number of SNPs tested in the GWAS; this threshold allowed one false positive association per genome scan. The genome-wide significance level was set to 0.05/N_snp_.

To identify genetic variants associated with strong sexual dimorphism, we tested for sex-specific differences in SNP effects using an approach similar to the one described in [6]. Briefly, we calculated the z-statistics as follows:

$$ Z=\frac{\beta_{male}-{\beta}_{female}}{\sqrt{se{\left({\beta}_{male}\right)}^2+se{\left({\beta}_{female}\right)}^2}}, $$

where *β*_*male*_ and *β*_*female*_ are the SNP effects estimated in males and females, respectively, and *se(β*_*male*_*)* and *se(β*_*female*_*)* are the standard errors of those SNP effects. Given the null hypothesis that SNP effects in males and female were equal, the z-statistic has a standard normal distribution. Single-tailed tests were used to evaluate the significance of any sex-specific difference. The significance level was set using the Bonferroni approach described above. To avoid spurious signals caused by low minor allele frequencies (MAF) in either sex, we exclude SNPs with MAF values of less than 5% (in males or females).

## Results

### Effect of sex on the traits studied

**Figure 1:**
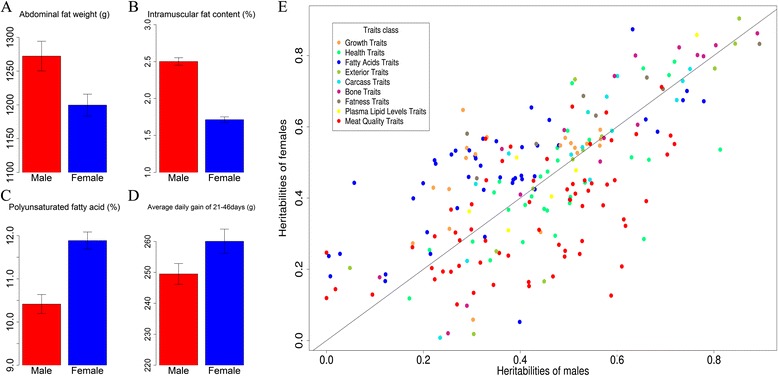
**Effect of sex on the phenotypic values and heritability of the 213 traits examined in this study. (A-D)** Four selected traits for which phenotypic values significantly differed between males and females. The error bars denote the standard errors associated with the average phenotypic values in either sex. **(E)** Comparison of the heritability of the 213 traits in males and females. Each dot represents a trait, and colors of the dots denote the trait categories.

We used a simple linear regression model to look for sex-specific differences in phenotypic values. Of the 213 traits examined, 102 showed significant sex-specific differences (q value [22] <0.05). Out of these 102 traits, 20 had q values greater than 1 × 10^−4^; these traits were related to fatness, fatty acid composition, growth and meat pH values (See Additional file 2: Table S2). Generally, males had greater abdominal fat weight (Figure 1A), greater intramuscular fat content (Figure 1B), greater leaf fat weight and higher levels of saturated fatty acids in both their fat and muscle tissues. Abdominal fat weight appeared to be the most significant sexually dimorphic trait (q value = 7.9 × 10^−13^); on average, males had 72 g more abdominal fat than did females (Figure 1A). Moreover, the meat of male pigs had a higher pH. In contrast, females had higher levels of unsaturated fatty acids and a higher average daily gain from 21 to 46 days old (q value = 1 × 10^−5^) (Figure 1C and D). Males and females showed a range of differences (from 0.74 to 1.58 fold) in their phenotypic values; most traits differed by more than 50% between the two sexes (See Additional file 2: Table S2).

### Heritability in males versus females

We also estimated the heritability of the 213 traits examined using separate male and female kinship matrices constructed using the autosomal 60 K SNP genotype data (see Methods section). Trait heritability was significantly correlated between males and females (Spearman correlation r = 0.65, *P* = 3.94 × 10^−27^) (See Additional file 3: Table S3) (Figure 1E). We found 24 traits that showed significant sex-specific differences in heritability (empirical *P* value <0.05). For instance, meat quality, forward gait score at 223 days old, platelet distribution width and serum glucose levels were more heritable in males than in females (See Additional file 3: Table S3) (Figure 1E). In contrast, fatty acid composition, weight at 46 days old and skin thickness were more heritable in females (See Additional file 3: Table S3) (Figure 1E). A trait with greater heritability is a trait for which a greater degree of phenotypic variance can be accounted for by genetic variants. This result indicates the importance of the sexually dimorphic genetic architecture that underlies these traits in pigs.

### Identifying sexually dimorphic loci associated with phenotypic traits

**Figure 2:**
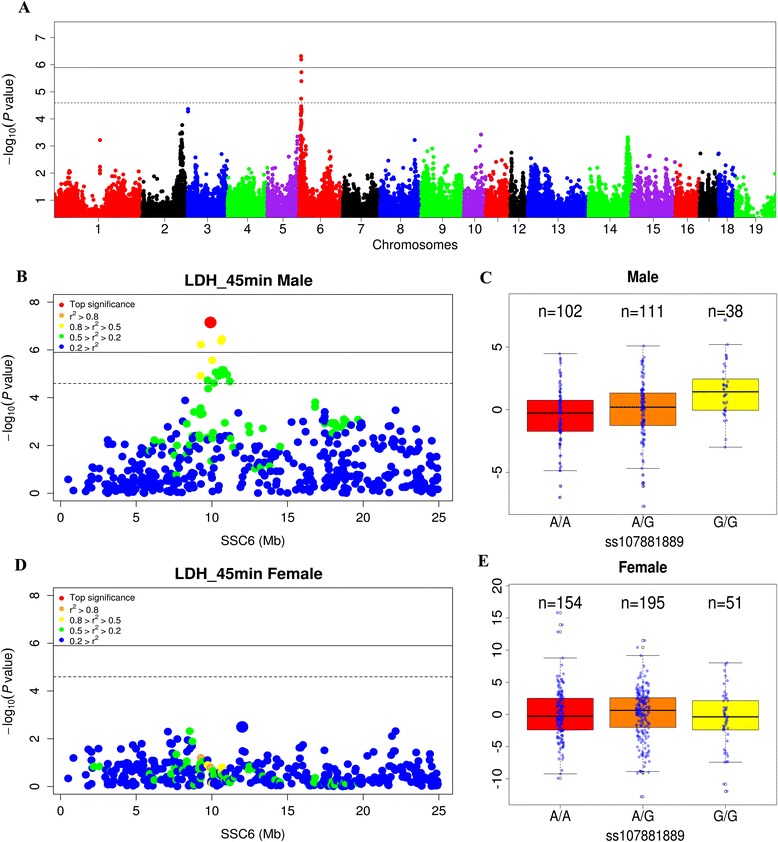
**Identification of sexually dimorphic loci using a sex-stratified GWAS. (A)** Manhattan plot of sex-specific signals; the significance of the sex-specific effects (−log_10_ *P* value) were plotted against the loci’s genomic positions. The dashed and solid horizontal lines represent suggestive and genome-wide significance thresholds. **(B)** and **(D)** Regional plots of GWAS signals on chromosome 6 for males **(B)** and females **(D)**. **(C)** and **(D)** Distribution of phenotypic values for the three SNP genotypes most associated with the *longissimus* muscle hue angle values in males **(C)** and females **(E)**.

To investigate the genetic basis of sexual dimorphism of traits, we performed a sex-stratified GWAS and assessed sex-specific differences in the allelic effects at each SNP locus using z-statistics [6] (see Methods section); this analysis revealed the extent of sex-specific genetic effects of particular SNPs on target traits. A total of 22 traits related to meat quality, fatty acid composition, hematological parameters and food intake were controlled by 43 significant (*P* <2.5 × 10^−5^) sexually dimorphic loci (See Additional file 4: Table S4). Of these 43 loci, 41 (95.3%) were on autosomes. The most significant signatures (*P* = 4.8 × 10^−7^) were detected for hue angle (H*) value at 45 minutes post-slaughter (Figure 2A). Hue angle is a meat color parameter related to Minolta b* (yellowness) and a* (redness) values, which can be calculated as H* = arctan(b*/a*). This locus exhibited a significant male-specific association (Figure 2B) but negligible association in females (Figure 2D). It should be noted that this locus was not detected in the GWAS that included both males and females (combined GWAS). This locus also showed a male-specific association with Minolta a* at 45 minutes post-slaughter, which is another meat redness parameter. Other notable traits associated with significant sexually dimorphic loci include stearic acid (C18:0) levels in muscle, food intake and mean corpuscular hemoglobin concentrations (see Additional file 4: Table S4).

**Figure 3:**
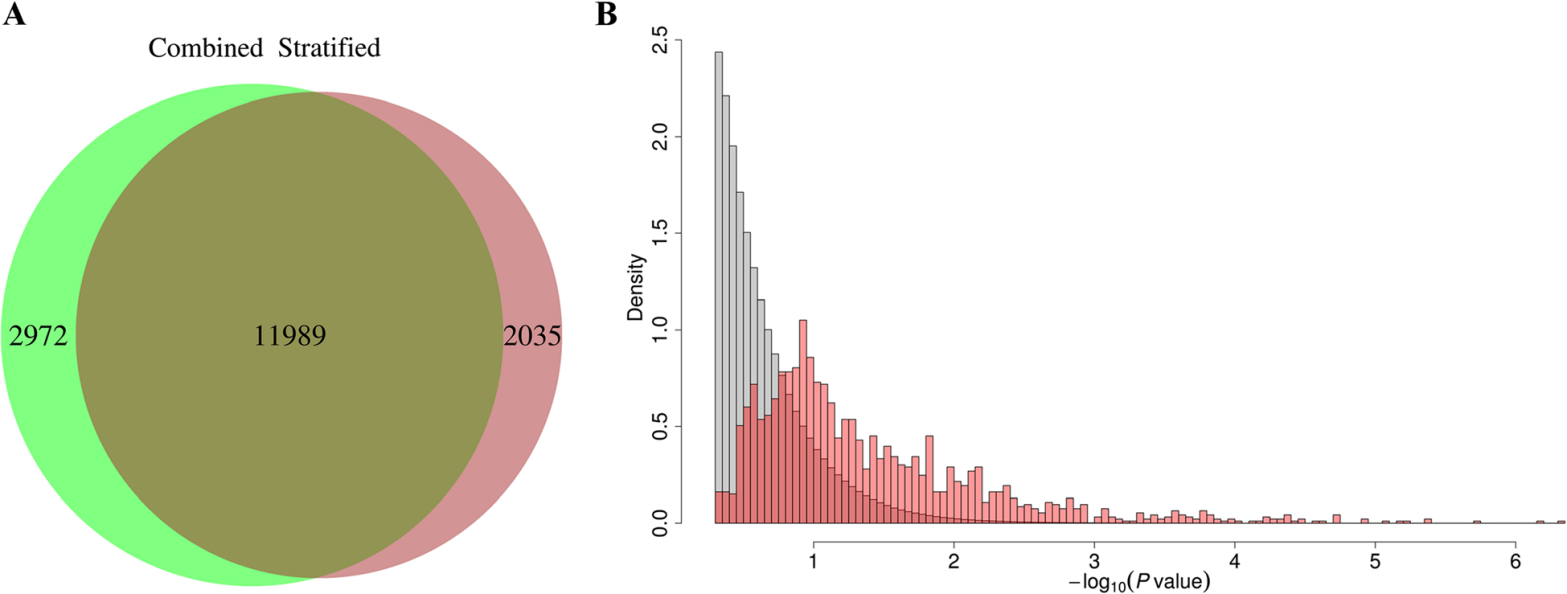
**Comparison of significant loci identified by the sex-stratified GWAS and the combined GWAS (which included both males and females).** **(A)** Venn diagram of the number of significant loci detected by the sex-stratified GWAS (red) and the combined GWAS (green). **(B)** Comparison of the distribution of the sex-specific signals (significance of the z- statistics) of all the SNPs and the 2035 additional significant trait-associated SNPs detected by the sex-stratified GWAS.

We compared the significant signals obtained in the sex-stratified GWAS with the significant signals obtained in the combined GWAS. Between the sex-stratified GWAS and the combined GWAS, we identified a total of 16 996 significant SNPs (*P* value <2.5 × 10^−5^) associated with the 213 traits. Of these SNPs, 70.5% were detected by both approaches. Notably, 2035 significant SNPs were detected only by the sex-stratified GWAS; this corresponds to 13.6% of the loci detected by the combined GWAS (Figure 3A). As a consequence, this result suggests that 13.6% more loci can be identified by using a sex-stratified GWAS in addition to a combined GWAS. We found that the values of the z-statistics associated with the new SNPs revealed by the sex-stratified GWAS were located in the tail of z-statistic distribution (Figure 3B) (*t* test *P* value = 2.3 × 10^−221^). This finding indicates that most of these new SNPs have sex-specific effects. Therefore, their signatures were weakened in the combined GWAS.

## Discussion

GWAS have become a powerful tool for detecting genetic variants associated with complex traits in pigs. Although studies in humans and model organisms have demonstrated the importance of sexual dimorphism in the genetic architecture of complex traits, to date nearly all the GWAS performed in pigs have ignored sex-specific effects or sex-by-genotype interactions, which means that they have possibly missed an opportunity to identify genetic variants that act differently in males and females.

To study sex-specific genetic effects on complex traits in pigs, we conducted a sex-stratified GWAS using 921 (507 males +414 females) F_2_ pigs produced by a White Duroc × Erhualian F_2_ cross. In contrast to human GWAS, the pigs in this GWAS were raised under the same controlled conditions and were fed a uniform diet; furthermore, most traits were phenotypically characterized at the same time (after slaughter). These experimental features have largely reduced the level of environmental noise and increased the ability to detect sexually dimorphic loci. Indeed, we were able to identify many extremely significant loci associated with traits related to meat quality [16], fatty acid composition [14], hematological parameters [17] and blood lipid levels [13], which suggests that the data obtained in this study can contribute to the identification of sex-specific genetic effects on complex traits.

Sex hormones are important factors that mediate sexually dimorphic gene expression in mice [23]. It should be noted that all the male pigs in this study were castrated when they were 90 days old. Therefore, the sex-specific effects identified in this study are less likely to have been caused by gonadal hormones than by sex chromosome dosage compensation or other unknown factors.

Of the 213 traits examined, 102 demonstrated significant sex-specific differences. Some of these traits were related to growth, fatness, meat quality, serum lipid levels and hematological parameters. These results indicate that sex is an important factor that affects the phenotypic values of various traits in pigs, even in the absence of the influence of sex hormones. Some traits showed a highly significant degree of sexual dimorphism (q value <10^−4^); for instance, we found that the castrated males studied in our study tended to accumulate more abdominal fat, leaf fat and intramuscular fat and that they had higher levels of saturated fatty acids in their fat and muscle tissue than females. From the standpoint of food quality, this result indicates that pork from castrated male pigs might taste better since IMF content is related to meat juiciness and flavor. In contrast, females grew faster during the early stages of life (from 21 to 46 days) and had higher levels of unsaturated fatty acids in their tissues; this result indicates that pork from female pigs might be healthier since foods containing a higher proportion of unsaturated fatty acids are better for human health [24]. These results support that sex does influence a broad range of traits in pigs.

Using genome-wide SNP genotypes to estimate the heritability of complex traits has proven to be feasible [25]. Furthermore, genetic component analyses using pedigree data have illustrated that significantly different types of genetic architectures underlie multiple traits related to body composition in humans [26]. However, few studies have examined differences in the heritability of complex traits between males and females using genotype data. Here, we estimated trait heritability separately in males and females using 60 K SNP genotypes. We found that the heritability of 22 traits differed significantly between males and females; some of these traits are related to waist vertebrae number, growth at an early age, platelet distribution width and serum glucose levels. Remarkably, we also found clusters of traits related to fatty acid composition and meat quality. Setting statistical significance aside, we found that SNP-genotype-based heritability was higher in females than in males for 35 (79.5%) of the 44 traits related to the fatty acid composition of fat or muscle tissues and for all of the fatness traits (Figure 1E) (see Additional file 3: Table S3). This finding indicates that genetic variants accounted for more of the variance in traits related to fatty acid composition and fatness in females than in males. In contrast, the heritability of most of the meat quality traits including temperature, pH, and the color of *longissimus* muscle and *semimembranosus* muscle at various time points post-slaughter was higher in males than in females (Figure 1E) (see Additional file 3: Table S3). Although these results could be biased by the presence of correlations between traits, they nonetheless illustrate that the genetic architecture that underlies certain categories of traits differed between the two sexes. This is possibly due to different evolutionary pressures imposed by natural and artificial selection that act to alter the molecular pathways that shape these traits in males versus females.

Compared to the combined GWAS (which included both males and females), the sex-stratified GWAS identified 13.6% more loci associated with the traits. This percentage of novel loci identified by the sex-stratified GWAS is similar to what has been found in studies of gene expression traits in humans [5]. This result illustrates the importance of performing sex-stratified GWAS or including a sex-by-genotype interaction in routine GWAS.

To identify sexually dimorphic loci associated with the traits being examined, we scanned the whole genome to compare the effects of SNPs in the two sexes. The most significant sexually dimorphic loci were found between 9.3 and 10.7 Mb on chromosome 6; they were associated with hue angle and Minolta a* values at 45 min post slaughter, which are color parameters that reflect meat redness. Meat color is an important feature for consumers since it reflects meat quality and freshness. It has been reported that ~90% of the variance in the color parameters of *longissimus* muscle in pigs can be explained by pigment content and myoglobin forms [27]. Moreover, the Minolta a* value has been reported to be significantly correlated with myoglobin expression in muscle in Pietrain pigs [28]. Presumably, the underlying genetic variants could affect pigment content or myoglobin content, which in turn could affect meat color. Using UCSC online gene annotation tools, we searched for candidate genes in a 1-Mb region around the SNPs that were significantly associated with hue angle values; the *nudix (nucleoside diphosphate linked moiety X)-type motif 7* gene (*NUDT7*), located at 10.1 Mb on chromosome 6 is of particular interest. This gene plays an important role in heme biosynthesis, and overexpression of *NUDT7* has been associated with reduced heme biosynthesis in pig skeletal muscle [29]. In mice, the hypomethylation of *NUDT7* in female offspring can be induced by high levels of maternal folic acid during gestation [30], which indicates that sex-specific epigenetic modification of this gene might be responsible for the sex-specific signals detected by the GWAS.

We found that 41 (95.3%) of the 43 significant sexually dimorphic loci were autosomal. This result is not unexpected since many health-related traits, such as fat distribution in humans, have also been observed to be associated with sexually dimorphic loci located on autosomes [31]. The molecular mechanisms that underlie these autosomal sexually dimorphic loci are not clear. Possible explanations are that sex-specific effects could contribute to heritable epigenetic differences between males and females [32] or that these differences are mediated by the different sex chromosomes found in males versus females.

We checked whether the 22 traits that were associated with significant sexually dimorphic loci demonstrated significantly different phenotypic values for males versus females. We found that 12 (54.4%) of the 22 traits did not have sex-specific phenotypic values (q >0.05). However, not one of the top five traits i.e. abdominal fat weight; C16:1, C18:0 and C20:3 levels in abdominal fat; and the saturated fatty acid content of muscle that demonstrated sex-specific phenotypic values (q <10^−4^) were associated with significant sexually dimorphic loci. These results suggest that even if phenotypic values differ significantly between the two sexes, it does not necessarily mean that strong sex-specific genetic controls are in place, and vice versa. Similar results have been reported for gene expression traits in humans [5].

## Conclusions

In this study, we looked for evidence of sexual dimorphism in the phenotypic values of and the genetic variants associated with 213 traits related to a broad range of pig traits. Some of these traits, namely those related to growth, fatness, meat quality and palatability, are of economic importance. The results of this study have improved our understanding of the relationship between sex and complex traits. About half of the 213 traits examined in this study showed significant sex-specific differences in their phenotypic values, which demonstrates that sex is an important factor that influences the phenotypic values of quantitative traits in pigs even when the influence exerted by sex hormones is limited. The sex-stratified GWAS revealed the existence of a landscape of sex-specific genetic effects on a variety of pig traits and demonstrated that sex-specific regulation is an important aspect of the genetic architecture that underlies complex traits. Our results suggest that future GWAS should stratify their analyses by sex or incorporate a sex-by-genotype interaction to identify sexually dimorphic loci associated with complex traits in pigs and other organisms.

## Additional files

Additional file 1: Table S1. Title: Descriptive statistics for the 213 traits examined in this study. Description: This file contains the abbreviation and description of 213 traits, and their descriptive statistics including mean, standard deviation, sample size and estimated heritability. Additional file 2: Table S2. Title: The 102 traits in this study that displayed significant sex-specific phenotypic values (q <0.05). Description: This file provides detailed statistical results on 102 traits that displayed sex dimorphic phenotype values. Additional file 3: Table S3. The sex-specific differences in the estimated heritabilities of the 213 traits examined in this study. Description: This file lists the estimated heritabilities of 213 traits for males and females, absolute difference between heritabilities of males and females, 5% significance thresholds of absolute difference between heritabilities of males and females obtained by 1000 permutation test. Additional file 4: Table S4. Significant sexually dimorphic loci associated with 24 traits identified in this study. Description: This file contains detailed information on 43 significant sexually dimorphic loci for 24 traits.
